# COVID-19: a risk factor for fatal outcomes in patients with comorbid cardiovascular disease

**DOI:** 10.18632/aging.103944

**Published:** 2020-10-09

**Authors:** Hui Xu, Ling Ai, Chun Qiu, Xi Tan, Bo Jiao, Ailin Luo, Shusheng Li, Shangkun Liu, Li Yan

**Affiliations:** 1 Department of Anesthesiology, Tongji Hospital of Tongji Medical College, Huazhong University of Science and Technology, Wuhan 430030, China; 2 Lazaridis School of Business and Economics, Wilfrid Laurier University, Waterloo N2L3C5, Canada; 3 Department of Emergency, Tongji Hospital of Tongji Medical College, Huazhong University of Science and Technology, Wuhan 430030, China

**Keywords:** cardiovascular disease, COVID-19, fatal outcome, corticosteroids

## Abstract

Objectives: To evaluate the fatal impact of COVID-19 on patients with comorbid cardiovascular disease (CVD).

Results: Overall, the 28-day mortality of patients with comorbid CVD was 3.25 times of that of patients without comorbid CVD (40.63% vs 12.50%, P=0.011). Clinic symptoms on admission were similar for the two groups. However, patients with comorbid CVD had higher levels of Interleukin-10 (22.22% vs 0%, P=0.034), procalcitonin (22.6% vs 3.13%, P<0.001), high-sensitivity troponin I (20 pg/mL vs 16.05 pg/mL, P=0.019), and lactic dehydrogenase (437 U/L vs 310 U/L, P=0.015). In addition, patients with comorbid CVD experienced a high incidence of acute respiratory distress syndrome (59.38% vs 15.63%, P<0.001), and required more invasive mechanical ventilation (40.63% vs 12.50%, P=0.011). Methylprednisolone was found to improve the survival of patients without comorbid CVD (p = 0.05).

Conclusions: Comorbid CVD resulted in a higher mortality rate for COVID-19 patients. Acute respiratory distress syndrome was the primary reason of death for COVID-19 patients with comorbid CVD, followed by acute myocardial infarction.

Methods: This retrospective study used propensity score matching to divide 64 COVID-19 patients into two groups with and without comorbid CVD. Clinic symptoms, laboratory features, treatments, and 28-day mortality were compared between the two groups.

## INTRODUCTION

The coronavirus disease 2019 (COVID-19), caused by severe acute respiratory syndrome coronavirus 2 (SARS-CoV-2), rapidly developed into a pandemic. By July 1^st^ 2020, there were more than 10 million people infected worldwide. A large proportion of those COVID-19 patients had pre-existing cardiovascular diseases (hereafter, CVD) [1]. A recent study on 1,099 COVID-19 patients reported that there were 14.9% patients with hypertension, and 2.5% with coronary heart disease [2]. The report from Chinese CDC, including 44,672 patients, showed that 4.2% accompanied CVD, and 12.8% had hypertension [3]. It also presented that 22.7% of fatal cases had comorbid CVD [3].

Previous studies indicated that acute respiratory infection posed a greater danger to CVD patients. Some researchers showed that among COVID-19 patients, cardiac injury and myocarditis were strong and independent factors associated with mortality, accompanied with an increase of troponin and a higher incidence of heart failure [4, 5]. Yet, what remains unclear is the fatal impact of COVID-19 on patients with comorbid CVD. What is also unknown is the effectiveness of treatments on COVID-19 patients with comorbid CVD. This study intends to address the questions above by investigating the association of comorbid CVD with the mortality of COVID-19 patients.

## RESULTS

### Demographics and baseline characteristics on admission

Out of 525 patients treated in the general wards and the intensive care unit (ICU) of Tongji Hospital, we selected 107 patients (56 males, 51 females) according to the selection criteria at the time of admission. Propensity score matching of age and gender resulted in the final selection of 64 patients enrolled in this study, with 32 patients in each group (CVD vs non-CVD). 36 (56.25%) patients were males and the remaining 28 were females (43.75%). The mean age was 61.53 [SD, 12.56] years. (Table 1)

**Table 1:** Baseline data of patients with COVID-19 before and after matching.

	**Without PS matching**				**With PS matching**			
	**Total (N=107)**	**with CVD (n=34)**	**Without CVD (n=73)**	***P***	**Total (N=64)**	**CVD group (n=32)**	**non-CVD group (n=32)**	***P***
**Age, mean (SD), y**	58.55±14.36	63.26±14.05	56.36±14.09	0.020	61.53±12.56	61.97±13.43	61.09±11.82	0.783
**Sex (n, %)**			0.080				0.313
Male	56(52.34)	22(64.71)	34(46.58)		36(56.25)	20(62.50)	16(50.00)	
Femal	51(47.66)	12(35.29)	39(53.42)		28(43.75)	12(37.50)	16(50.00)	
**Smoking (n, %)**	26(24.30)	6(17.65)	20(27.40)	0.274	11(17.19)	6(18.75)	5(15.63)	0.740

**Abbreviations: SD, standard deviation**

### Clinic symptoms on admission

On admission, none of the 64 patients showed evidence of acute myocardial infarction, thromboembolic disease, chronic liver disease, chronic kidney disease, or rheumatism. 58 (90.63%) patients had fever, 40 (62.50%) had cough, 25 (39.06%) had dyspnea, 24 (37.50%) had sputum production, and 22 (34.38%) exhibited chest tightness. Eight (12.50%) patients had only one symptom on admission while 34 (53.13%) had more than four symptoms. There was no statistical difference in age, gender, symptoms, temperature, heart rate and respiratory rate between the two groups (Table 2). Based on the sample, it seems that underlying CVD conditions did not influence the symptoms, heart rate, breath rate, and temperature of COVID-19 patients. However, the mean blood pressure in the CVD group was 100.30 [17.67] mmHg, which was higher than that of the non-CVD group (91.08 [8.63] mmHg). There were 11 (34.38%) general cases, 11 (34.38%) severe cases, and 10 (31.25%) critical cases in the CVD group. There were 22 (68.75%) general cases, 10 (31.25%) severe cases, but no critical cases in the non-CVD group (P=0.001).

**Table 2:** Demographics and clinical characteristics of patients with COVID-19 on admission.

	**Total**	**CVD group**	**non-CVD group**	***P***
	**(N=64)**	**(n=32)**	**(n=32)**	
**Comorbidities (n, %)**				
Hypertension	14(43.75)	14(43.75)	0	
CHD	8(25.00)	8(25.00)	0	
Hypertension +CHD	10(31.25)	10(31.25)	0	
**Symptoms (n, %)**				
Fever	58(90.63)	29(90.63)	29(90.63)	>0.999
Cough	40(62.50)	20(62.50)	20(62.50)	>0.999
Fatigue	18(28.13)	8(25.00)	10(31.25)	0.578
Anorexia	10(15.63)	6(18.75)	4(12.50)	0.491
Myalgia	13(20.31)	4(12.50)	9(28.13)	0.120
Dyspnea	25(39.06)	13(40.63)	12(37.50)	0.798
Chest tightness	22(34.38)	14(43.75)	8(25.00)	0.114
Sputum production	24(37.50)	10(31.25)	14(43.75)	0.302
Hemoptysis	1(1.56)	0(0)	1(3.13)	>0.999
Pharyngalgia	3(4.69)	1(3.13)	2(6.25)	>0.999
Diarrhea	17(26.56)	7(21.88)	10(31.25)	0.396
Nausea and Vomiting	8(12.50)	4(12.50)	4(12.50)	>0.999
Abdominal pain	3(4.69)	1(3.13)	2(6.25)	>0.999
Headache	7(10.94)	3(9.38)	4(12.50)	>0.999
Dizziness	5(7.81)	2(6.25)	3(9.38)	>0.999
Disorders of consciousness	1(1.56)	1(3.13)	0(0)	>0.999
Shortness of breath	11(17.19)	7(21.88)	4(12.50)	0.320
Chest pain	4(6.25)	1(3.13)	3(9.38)	0.606
Multiple symptoms				
1 symptom	8(12.50)	5(15.63)	3(9.38)	0.705
2 symptoms	9(14.06)	5(15.63)	4(12.50)	>0.999
3 symptoms	13(20.31)	6(18.75)	7(21.88)	0.756
≥4 symptoms	34(53.13)	16(50.00)	18(56.25)	0.616
**Vital Signs**				
Body temperature, median (IQR), °C	37.40(36.80,38.00)	37.50(36.68,38.00)	37.30(36.80,38.00)	0.803
Heart rate, mean (SD), bpm	94.77±14.43	96.94±12.84	92.59±15.78	0.232
Respiratory rate, median (IQR), per min	20.00(20.00,23.00)	20.00(20.00,28.75)	20.00(20.00,21.75)	0.782
Mean blood pressure, mean (SD), mmHg	95.69±14.56	100.30±17.67	91.08±8.63	0.011
**Venous thromboembolism risk (Caprini Risk Score)**			0.462
Low risk, n/N, (%)	5(7.81)	3(9.38)	2(6.25)	
Moderate risk, 2,n/N, (%)	16(25.00)	9(28.13)	7(21.88)	
High risk, n/N, (%)	35(54.69)	18(56.25)	17(53.13)	
Highest risk, n/N, (%)	8(12.50)	2(6.25)	6(18.75)	
**Classification of severity of COVID-19(n, %)**				0.001
general cases	33(51.56)	11(34.38)	22(68.75)	0.006
severe cases	21(32.81)	11(34.38)	10(31.25)	0.790
critical cases	10(15.63)	10(31.25)	0(0)	0.001

Abbreviations: SD, standard deviation; IQR, interquartile range; CHD, coronary heart disease

### Laboratory results on admission

Patients with comorbid CVD presented with significantly higher white blood cell count (median [IQR], 7.27 [4.79-9.31] vs 5.27 [3.59-6.52] /μL [to convert to ×10^9^per liter]); P=0.016) than those in the non-CVD group. Patients with comorbid CVD also had significantly higher Interleukin-10 (IL-10) and procalcitonin (PCT) on admission (P=0.034 and P<0.001, respectively). Specifically, 7 (22.58%) patients in the CVD group presented higher PCT exceeding 0.5ng/ml, while only 1 (3.13%) patient’s PCT reached this level in the non-CVD group. According to IL-10, 6 (22.20%) patients accompanied with IL-10 more than 9.1pg/ml in the CVD-group; however, IL-10 of the patients in the non-CVD group never exceeded 9.1pg/ml. Although the level of high sensitivity troponin I (hs-cTnI) on admission was generally high in both groups, the patients in the CVD group still presented a significantly higher hs-cTnI level than those in the non-CVD group (median [IQR], 20.00 [16.60-25.95] vs. 16.05 [6.55-21.63] pg/mL, P=0.019). Additionally, patients with CVD presented with a significantly higher level of lactate dehydrogenase (LDH) (median [IQR], 437 [308.00-581.00] vs 310 [252.00-446.00] U/L; P=0.015) than those without CVD. Patients in the CVD group also showed a significantly higher level of potassium (median [SD], 4.40 [0.57] vs 4.11 [0.51] mmol/L; P =0.035), and blood urea nitrogen (BUN) (median [IQR], 6.50 [3.50-9.50] vs 4.05 [2.85-5.25] mmol/L; P=0.010). The other laboratory findings were similar between the two groups (Table 3).

**Table 3:** Laboratory features on admission.

	**Total**	**CVD group**	**Non-CVD group**	***P***
	**(N=64)**	**(n=32)**	**(n=32)**	
**Blood Routine Test**				
White blood cell count, median (IQR), (n), ×10^9^/L	5.70(4.23,7.95),(63)	7.27(4.79,9.31),(31)	5.27(3.59,6.52),(32)	0.016
Lymphocyte percentage, mean (SD), (n),%	16.63±9.53,(63)	14.38±9.43,(31)	18.80±9.26,(32)	0.066
Lymphocyte count, mean (SD)(n),×10^9^/L	0.89±0.47,(63)	0.88±0.52,(31)	0.90±0.43(32)	0.877
Hematocrit, mean (SD), (n), %	37.08±5.09,(63)	36.89±5.74,(31)	37.26±4.46,(32)	0.774
**Blood Bio-Chemistry Test**				
ALT, median (IQR), (n), U/L	30.50(20.75,49.00),(62)	33.00(21.00,57.00),(31)	29.00(19.00,46.00),(31)	0.288
Albumin, mean (SD), (n), g/L	31.96±4.70,(62)	31.39±4.18,(31)	32.53±5.17,(31)	0.344
Cystatin C, median (IQR), (n), mg/L	0.94(0.81,1.17),(32)	1.02(0.82,1.22),(15)	0.90(0.80,0.97),(17)	0.141
Blood glucose, median (IQR), (n), mmol/L	6.48(5.65,8.82),(63)	6.55(5.77,10.22),(31)	6.37(5.65,8.31),(32)	0.587
eGFR, mean (SD), (n), ml/min/1.73m^2^	85.42±22.67,(63)	80.12±25.58,(31)	90.56±18.42,(32)	0.067
Creatinine, median (IQR), (n), μmol/L	69.00(52.00,94.00),(63)	78.00(58.00,101.00),(31)	66.00(50.50,90.75),(32)	0.099
BUN, median (IQR), (n), mmol/L	4.50(3.50,7.60),(63)	6.50(3.50,9.50),(31)	4.05(2.85,5.25),(32)	0.010
K^+^, mean (SD), (n), mmol/L	4.25±0.55,(62)	4.40±0.57,(30)	4.11±0.51,(32)	0.035
Na^+^, mean (SD), (n), mmol/L	138.46±4.22,(62)	139.19±4.72,(30)	137.77±3.62,(32)	0.189
Cl^-^, mean (SD), (n), mmol/L	100.51±3.86,(62)	101.01±4.33,(30)	100.04±3.35,(32)	0.327
Ca^+^, median (IQR), (n), mmol/L	2.12(2.03,2.16),(62)	2.08(1.97,2.17),(30)	2.13(2.04,2.16),(32)	0.535
HCO_3_^-^, median (IQR), (n), mmol/L	22.70(21.10,24.10),(63)	22.70(20.90,24.00)(31)	22.90(21.55,24.30),(32)	0.554
**Coagulation Function Test**				
PT, median (IQR), (n), s	14.35(13.78,15.33),(62)	14.40(13.80,16.20),(31)	14.30(13.50,15.00),(31)	0.281
KPPT, mean (SD), (n), s	39.99±6.96,(57)	40.33±7.57,(30)	39.62±6.34,(27)	0.702
D-dimer,μg/mL				0.668
<0.5,n/N,(%)	8/61(13.11)	5/30(16.67)	3/31(9.68)	
≥0.5,n/N,(%)	53/61(86.89)	25/30(83.33)	28/31(90.32)	
**Infection-Related Bio-Markers**				
Ferritin, median (IQR), (n), μg/L	770.20(561.50,1202.80), (45)	940.90(713.15,1462.20),(21)	625.10(504.15,1203.65),(24)	0.053
High sensitivity C-reactive protein, median (IQR), (n), mg/L	57.90(27.73,98.85),(62)	69.10(31.25,111.48),(30) (30)	54.90(27.18,87.30),(32)	0.338
procalcitonin, ng/mL				<0.001
0.02-0.05, n/N, (%)	15/63(23.81)	1/31(3.23)	14/32(43.75)	
<0.02, n/N, (%)	3/634.76)	3/31(9.68)	0/32(0)	
0.05-0.5, n/N, (%)	37/63(58.73)	20/31(64.52)	17/32(53.13)	
0.5-2, n/N, (%)	5/63(7.94)	5/31(16.13)	0/32(0)	
≥2, n/N, (%)	3/63(4.76)	2/31(6.45)	1/32(3.13)	
Erythrocyte sedimentation rate, median (IQR), (n), mm/h	37.00(22.25,59.75),(58)	37.50(9.00,62.00),(26)	37.00(28.00,47.75),(32)	0.673
IL-1β, pg/mL				0.127
<5, n/N, (%)	44/53(83.02)	25/27(92.59)	19/26(73.08)	
≥5, n/N, (%)	9/53(16.98)	2/27(7.41)	7/26(26.92)	
IL-2R, median (IQR), (n), U/mL	774.00(564.50,1273.00), (63)	838.00(606.00,1511.00),(27)	723.00(540.50,1013.50),(26)	0.188
TNF-a, median (IQR), (n), pg/mL	8.70(7.30,11.85),(63)	11.30(7.50,13.70),(27)	8.45(7.18,10.50),(26)	0.137
IL-6, pg/Ml				0.934
<7, n/N, (%)	14/53(26.42)	7/27(25.93)	7/26(26.92)	
≥7, n/N, (%)	39/53(73.58)	20/27(74.07)	19/26(73.08)	
IL-8, pg/mL				>0.999
<62, n/N, (%)	46/53(86.79)	23/27(85.19)	23/26(88.46)	
≥62, n/N, (%)	7/53(13.21)	4/27(14.81)	3/26(11.54)	
IL-10, pg/mL				0.034
<9.1, n/N, (%)	47/53(88.68)	21/27(77.78)	26/26(100)	
≥9.1, n/N, (%)	6/53(11.32)	6/27(22.22)	0/26(0)	
**Myocardial Injury Bio-Markers**				
LDH, median (IQR), (n), U/L	353.50(280.00,516.00),(62)	437.00(308.00,581.00),(31)	310.00(252.00,446.00),(31)	0.015
NT-proBNP, median (IQR), (n), pg/Ml	402.00(106.00,895.25),(40)	442.50(194.25,1562.25),(22)	159.50(65.75,695.00),(18)	0.109
ACE, mean (SD), (n), U/L	24.38±10.25,(8)	18.50±7.78,(2)	26.33±10.80,(6)	0.390
Hs-cTnl, median (IQR), pg/mL	18.10(8.60,23.28)	20.00(16.60,25.95)	16.05(6.55,21.63)	0.019

Abbreviations: IQR, interquartile range; SD, standard deviation; ALT, Alanine aminotransferase; GFR, Glomerular filtration rate; BUN, Blood urea nitrogen; PT, Prothombin time; KPTT, Activated partial thromboplastin time; LDH, Lactate dehydrogenase; NT-proBNP, amino-terminal pro-brain natriuretic peptide; ACE, Angiotension converting enzyme; Hs-cTnl, High sensitivity troponin I.

Data are mead±standard deviation, or median (IQR), n, or n/N (%), where N is the total number of patients with available data.

### Summary of treatments during hospitalization

For hospitalized patients, the main treatment approaches included antiviral (lopinavir 400mg/ritonavir 100mg twice daily; Arbidol 0.2g 3 times daily), antibacterial (moxifloxacin, 0.4g once daily), and glucocorticoid (methylprednisolone, 40mg once daily or 40mg twice daily) (Table 4). There was no significant difference in such therapies between patients with comorbid CVD and without CVD. However, the rate of deploying invasive mechanical ventilation was much higher in patients in the CVD group than in the non-CVD group (13 [40.63%] vs. 4 [12.50%], P = 0.011). The incidence of acute respiratory distress syndrome (ARDS) in the CVD group was significantly higher than that of COVID-19 patients without CVD (19 [59.38%] vs. 5 [15.63%], P < 0.001).

**Table 4:** Treatment and clinical outcomes of patients with COVID-19.

	**Total**	**CVD group**	**non-CVD group**	***P***
	**(N=64)**	**(n=32)**	**(n=32)**	
**Treatments (n, %)**				
Methylprednisolone	51(79.69)	25(78.13)	26(81.25)	0.756
Antivirus	51(79.69)	24(75.00)	27(84.38)	0.351
Antibiotic	58(90.63)	28(87.50)	30(93.75)	0.668
**Ventilation support (n, %)**			0.013
Oxygen therapy	39(60.94)	14(43.75)	25(78.13)	0.005
NIV	8(12.50)	5(15.63)	3(9.38)	0.450
IMV	17(26.56)	13(40.63)	4(12.50)	0.011
**Complications (n, %)**				
ARDS	24(37.50)	19(59.38)	5(15.63)	<0.001
AMI	5(7.81)	5(15.63)	0(0)	0.062
AKI	4(6.25)	3(9.38)	1(3.13)	0.606
Heart Failure	4(6.25)	4(12.50)	0(0)	0.121
**Clinical outcomes (n, %)**			0.011
Survived	47(73.44)	19(59.38)	28(87.50)	
Died	17(26.56)	13(40.63)	4(12.50)	
**Hospitalization stay, median (IQR), days**			
Hospital stay	28.54(17.57,35.32)	20.95(8.19,32.28)	30.56(28.53,36.66)	0.002
Hospital stay of dead cases	8.05(3.89,16.75)	7.10(3.89,16.09),(13)	11.34(3.80,27.67),(4)	0.497

Abbreviations: NIV, non-invasive ventilation; IMV, Invasive ventilation; ARDS, acute respiratory distress syndrome; AMI, acute myocardial infarction; IQR, interquartile range.

### The mortality outcomes and CVD

By the end of March 25, 2020, 17 (26.56%) patients died during hospitalization; 13 (40.63%) in the CVD group vs. 4 (12.50%) in the non-CVD group. The mortality rate in the CVD group was 3.25 times of that in the non-CVD group (95% CI 1.19-8.90). The patients in the CVD group had accordingly a shorter hospitalization time (median [IQR], 20.95 [8.19-32.28] vs 30.56 [28.53-36.66] days; P = 0.002) (Table 4).

**Figure 1:**
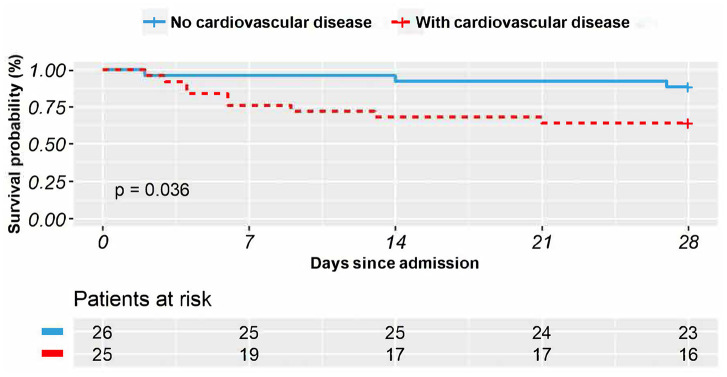
**The Kaplan–Meier survival curves in 28 days for COVID-19 patients who received methylprednisolone treatment with vs. without comorbid cardiovascular disease.**

Survival analysis was first conducted to compare the days patients lived during the timespan of the study; the results were depicted via two Kaplan-Meier plots. We first compared the survival status of patients treated with methylprednisolone to see whether methylprednisolone was particularly effective in treating one of the two groups. 25 patients in the CVD group with methylprednisolone treatment survived an average of 21 days (95% CI 17-25) during the 28 days of this study. In comparison, 26 patients in the non-CVD group with methylprednisolone treatment survived an average of 26 days (95% CI 24-29). The comparison results were plotted (Figure 1). The logrank test indicated that patients without CVD lived significantly longer than patients with CVD when methylprednisolone treatment was applied (P = 0.036).

**Figure 2:**
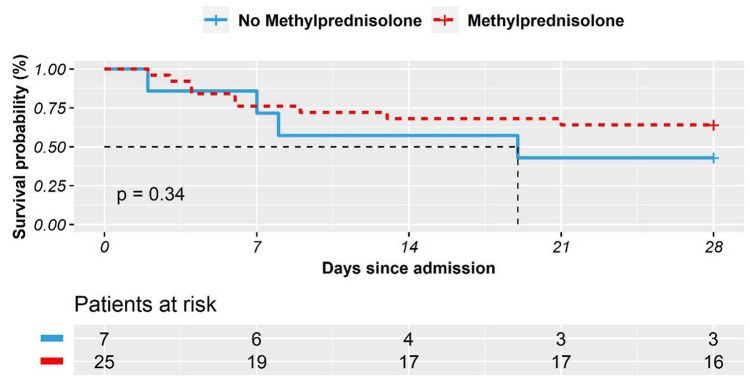
**The Kaplan–Meier survival curves in 28 days for COVID-19 patients with comorbid cardiovascular disease who received methylprednisolone treatment vs. who did not.**

We also compared the survival time of COVID-19 patients with CVD, treated with or without methylprednisolone. On average, 25 patients with CVD who received methylprednisolone treatment survived 21 days (95% CI 17-25). The seven patients in the CVD group who did not receive methylprednisolone treatment survived an average of 17 days (95% CI 9-25). The comparison results were plotted (Figure 2). The logrank test was not significant (P = 0.340).

### Survival and the associated factors

To formally examine the factors that were associated with the survival time of COVID-19 patients, we conducted a series of univariate cox regression analyses in the population. The univariate analyses revealed that CVD was to be associated with a worse prognosis in COVID-19 patients, so were the age and respiratory rate (Table 5). We also performed a multivariate cox regression analysis in the population, and found that higher mean blood pressure (MBP) was negatively associated with patients’ survival, whereas higher oxygen saturation (SpO_2_) level was positively associated with patients’ survival. After controlling for age, gender, and vital signs, we found that methylprednisolone treatment significantly decreased mortality in COVID-19 patients (P = 0.041).

**Table 5:** Univariate and multivariate cox regression analyses for COVID-19 patients.

**Variables**	**Univariate analysis**		**Multivariate analysis**	
	**HR (95% CI)**	**P**	**HR (95% CI)**	**P**
CVD	3.99 (1.30-12.26)	0.016	5.86 (1.55-22.13)	0.009
Methylprednisolone treatment	0.57 (0.20-1.62)	0.289	0.27 (0.08-0.95)	0.041
Gender	1.97 (0.69-5.58)	0.205	2.01 (0.65-6.23)	0.224
Age group	2.10 (1.19-3.69)	0.010	1.95 (1.17-3.24)	0.010
SpO_2_ (oxygen saturation)	0.96 (0.92-1.01)	0.089	0.91 (0.84-0.99)	0.020
Body temperature	0.70 (0.38-1.28)	0.246	0.56 (0.26-1.19)	0.132
Heart rate	1.03 (1.00-1.07)	0.078	1.02 (0.98-1.07)	0.322
Respiratory rate	1.10 (1.02-1.18)	0.011	0.27 (0.08-0.95)	0.041
Mean blood pressure	1.02 (0.99-1.05)	0.198	0.93 (0.87-0.99)	0.009

To further demonstrate the difference of methylprednisolone treatment between the two groups, a multivariate cox regression was conducted in the CVD group and the non-CVD group, respectively (Table 6). In the CVD group, the effect of methylprednisolone treatment was not statistically significant (P = 0.241). In comparison, in the non-CVD group, methylprednisolone treatment was found to reduce the hazard rate (P = 0.05). In the CVD group, oxygen saturation (SpO_2_) was linked to shorter survival time, while high respiratory rate was associated with shorter survival time in the non-CVD group. Age remained a significant factor associated with higher mortality in the CVD group, this was not the case in the non-CVD group.

**Table 6:** Multivariate cox regression for COVID-19 patients in CVD and non-CVD groups.

**Variables**	**CVD group**		**Non-CVD group**	
	**HR (95% CI)**	***p*-Value**	**HR (95% CI)**	***p*-Value**
Methylprednisolone treatment	3.99 (1.30-12.26)	0.241	0.00 (0.00-1.02)	0.050
Gender	0.57 (0.20-1.62)	0.792	0.06 (0.00-12.75)	0.307
Age group	1.97 (0.69-5.58)	0.016	55.49 (0.79-3885.13)	0.064
SpO_2_ (oxygen saturation)	2.10 (1.19-3.69)	0.007	1.11 (0.83-1.48)	0.497
Body temperature	0.96 (0.92-1.01)	0.127	0.00 (0.00-2.47)	0.089
Heart rate	0.70 (0.38-1.28)	0.282	1.02 (0.92-1.12)	0.731
Respiratory rate	1.03 (1.00-1.07)	0.140	1.70 (1.04-2.79)	0.036
Mean blood pressure	1.10 (1.02-1.18)	0.138	0.80 (0.63-1.03)	0.081

## DISCUSSION

To date, age (>60 years), gender (male), and the presence of comorbidities are believed to be the major risk factors for COVID-19 mortality [6, 7]. This study focused on the deterioration and mortality of COVID-19 patients with comorbid CVD, excluding other comorbidities. In addition, it took treatments into consideration. There were 24 (75% in CVD group) COVID-19 patients experiencing hypertension, among which ten (31.25%) patients also had CHD. The comorbidity composition was consistent with the literature that hypertension was the most common comorbidity [8, 9]. For patients with CVD, COVID-19 infection may either precipitate a myocardial infarction (Type one myocardial infarction,) increasing myocardial demand that leads to worsening ischemia and necrosis (Type two myocardial infarction), or directly increase metabolic demand that leads to heart failure and death [9].

This study found that there were no significant differences in clinical manifestations between patients with and without CVD upon admission. Patients in both groups demonstrated similar symptoms related to the respiratory system, including dry cough, dyspnea, sputum production, and chest tightness. The Caprini risk scores for the venous thromboembolism of the patients in both groups were also similar, and there was no evidence indicating that any patient enrolled in this study had thromboembolic disease upon admission. It is not surprising that on average patients in the CVD group had higher MBP, which may be due to the long-period elevated vascular resistance in comorbid CVD. Furthermore, according to the multivariate cox regression, a higher MBP was found to reduce COVID-19 patient survival time; and the patients with comorbid CVD exhibited a worse tolerance to hypoxia, which was characterized by a significant reduction in survival time.

This study identifies a number of significant differences between the two groups from illness onset: Patients with comorbid CVD are more likely to exhibit elevation of hs-cTnl levels (median [IQR], 20.00 [16.60-25.95] pg/mL) compared with patients without CVD (median [IQR], 16.05 [6.55-21.63] pg/mL). Current studies have shown that patients with comorbid CVD are more likely to experience myocardial injury and be at higher risk of death following COVID-19 infection. The potential mechanisms include direct damage by virus, systemic inflammatory response, and severe hypoxia [10]. In addition, this study confirms the findings in Shi et al. [11], which reported that patients with elevated hs-cTnI had higher leukocyte and PCT levels, but lower lymphocyte count.

The laboratory data in this study were based on the results of patient admission at hospital. At that time, the differences in PCT and IL-10 were substantial within the two groups, suggesting that patients in the CVD group had a higher risk of bacterial infection than those in the non-CVD group. Yet, the exact condition of bacterial infection was not confirmed. These results were consistent with previous studies suggesting that higher levels of infection-related bio-markers were associated with more severe inflammatory inducing organ damage and higher mortality in COVID-19 [8, 10].

In our study, the elevated level of LDH on admission was more common among COVID-19 patients with comorbid CVD. Particularly for severe COVID-19 patients, the increase of LDH is significant, and is one of the most important prognostic markers of organ injury and mortality [12]. Meanwhile, BUN and potassium levels were also significantly higher in the CVD group. Due to the retrospective study design, some laboratory tests, including N-terminal B-type natriuretic peptide (NT-proBNP), high sensitivity C-reactive protein (hs-CRP), and serum ferritin were not conducted on all the patients. Therefore, their role might be underestimated in evaluating their effect on prognosis in the COVID-19 patients with comorbid CVD.

On admission, the ratio of severe and critical cases in the CVD group (21/32, 65.63%) was significantly higher than that of the non-CVD group (10/32, 31.25%). There were ten cases classified as critical in the CVD group, who had more than ten years of hypertension combined with CHD, and who had presented with acute hypoxemic respiratory failure that required ventilator support. Accordingly, four of them received noninvasive ventilation (NIV); and six received IMV immediately. In comparison, there was no critical case in the non-CVD group on admission. Moreover, during hospitalization, patients in the CVD group were more susceptible to the development of ARDS, and hence were placed on more ventilation support than patients in the non-CVD group. Specifically, in the CVD group, five cases (15.63%) received NIV, and thirteen cases (40.63%) received IMV, which indicated the deterioration of lung function; in the non-CVD group only three patients (9.38%) received NIV, and four patients (12.50%) received IMV.

The need for NIV or IMV for critical patients has received increased attention among medical workers. The proportion of invasive ventilator support in ICU varied greatly from place to place: 88% in Lombardy, Italy [7], 71% in Washington State, US [13], 30%-47% in Wuhan, China [8, 14]. Conversely, in previous reports, NIV had been used more frequently for critical COVID-19 patients [14, 15]. In our study, the proportion of IMV support was 40.60% (13 of 32) in the CVD group, which was much higher than that of the non-CVD group (12.50%, 4 of 32), indicating that a comorbid CVD is a high-risk factor for critical illness in COVID-19. Unfortunately, none of the patients in our study who were treated with an invasive ventilator were saved. This mortality rate is much higher than that reported in a recent study [16], which was only 18% (6 of 34). The difference may be due to the effect of the use of remdesivir in that study. However, appropriate timing in the use of IMV for critical COVID-19 patients is still worthy of further investigation.

Until now, no specific medication has been recommended for treating COVID-19 except for symptomatic supportive treatment and intervention. In our study, most patients received antiviral, antibacterial, and glucocorticoid (methylprednisolone) therapy during hospitalization. The antiviral and antibacterial treatments did not make any significant difference between the two groups.

This study also identifies an interaction effect between methylprednisolone treatment and comorbid CVD (Figure 2). That is, the effectiveness of methylprednisolone treatment of COVID-19 was contingent on patients’ comorbid CVD. The multivariate cox regression further provided some implication of the use of methylprednisolone for COVID-19 patients. Specifically, when the effects of age, gender and vital signs were controlled, the methylprednisolone treatment was statistically insignificant for the patients with comorbid CVD (p = 0.241). Yet, the use of methylprednisolone treatment did not worsen patients’ condition either. In comparison, in the non-CVD group, methylprednisolone treatment was found to statistically improve the survival of COVID-19 patients. More rigorous research shall be conducted to address the effectiveness and the possible side effects [17, 18] of methylprednisolone treatment.

Finally, compared with the non-CVD group, COVID-19 patients with comorbid CVD were associated with a higher mortality rate (40.63% vs 12.50%) and accordingly experienced a shorter hospital stay (20.95 vs 30.56 days). The incidence and mortality of ARDS in patients with cardiac injury were higher than those without cardiac injury [19]. Our results were consistent with the popular notion that ARDS was the primary reason of death for COVID-19 patients with CVD, followed by acute myocardial infarction (AMI) [20]. Moreover, heart failure could be another major factor contributing to the fatality risk of COVID-19 patients with or without history of previous cardiovascular disease [21].

This study has several limitations. Firstly, only 64 patients with normal cardiac function were included. Whether cardiac dysfunction is associated with a higher COVID-19 mortality rate needs further discussion. In further studies, our sample size should be amplified. Secondly, some specific information from ICU is missing, such as mechanical ventilation settings. In the electronic medical records, progressive changes of the illness were recorded, whether the patient used a ventilator or progressed to ARDS, as well as the cause of death. However, the mechanical ventilation parameters of the ventilator were not fixed during the course of the illness and would be adjusted at any time according to the needs of the patient. There was no detailed record regarding the ventilation parameters in the electronic medical records. Thirdly, due to the extremely limited resources available at the beginning of the COVID-19 outbreak, we were unable to obtain complete medical information before admission for those patients whom we retrospectively studied. Hence, we were unable to assess the impact of angiotensin converting enzyme inhibitors/angiotensin receptor blockers (ACEIs/ARBs) on the prognosis of COVID-19 patients with comorbid CVD. Based on current data, there was no evidence that ACEIs or ARBs increased the risk of COVID-19 [22, 23]. For patients with COVID-19 who previously used ACEIs/ARBs, the use of these drugs may not need to be discontinued [1, 24].

In conclusion, COVID-19 severely challenged the survival of those patients with CVD who are prone to progression to severe or critical conditions. Comorbid CVD is associated with a higher mortality rate among COVID-19 patients. Age is also a factor in the mortality rate of patients with CVD. Some deteriorating vital signs are good indicators for mortality only for COVID-19 patients with CVD, which could provide some diagnostic value for physicians treating this type of patient. Conventional medical treatments were not associated with prognosis improvement, except methylprednisolone treatment which was found to be associated with the extended survival of COVD-19 patients without CVD. Nevertheless, such an effect shall be examined in a larger population to establish credible linkage for the usage of methylprednisolone.

## MATERIALS AND METHODS

### Study design and participants

This retrospective single-centered study was approved by the Human Assurance Committee (HAC) of Tongji Hospital (affiliated with Tongji Medical College, Huazhong University of Science and Technology, Wuhan, China). Consent was obtained from patients or patients’ next of kin. We retrospectively analyzed hospitalized COVID-19 patients in accordance with interim guidelines of the World Health Organization (WHO) [25] from January 28, 2020 to February 14, 2020, who were either discharged or deceased before March 25,2020.

### The selection criteria for cardiovascular diseases and data collection

All COVID-19 patients enrolled in this study had no comorbidities other than the comorbid CVD, which were defined as hypertension and coronary heart disease (CHD). Any patients with abnormal cardiac function upon admission were excluded from the analysis. Additionally, the patients who died on admission day to hospital, were excluded. Patients’ characteristics that were collected for analysis included demographics, comorbid CVD, laboratory examinations on admission, and treatments during hospitalization, including methylprednisolone.

### Outcome

The end point was the 28-day mortality associated with COVID-19. The clinical recovery and discharge criteria included remission of clinical symptoms, normal body temperature, obvious regression of inflammation in chest CT, and at least two consecutive negative results of SARS-CoV-2 detection by real-time reverse transcriptase polymerase chain reaction.

### Statistical methods

All continuous variables were described with mean [standard deviation] if they follow a normal distribution, or as quartiles if not. T test was applied to variables that fit a normal distribution. Non-parametric tests via Mann-Whitney U or Kruskal-Wallis tests were applied to variables that did not follow a normal distribution. Standard treatment of Chi-square test was applied to categorical variables depicted as counts or percentage.

To have an accurate comparison to net the effect of CVD condition on patients’ survival status, propensity score matching was used. Patients were selected by CVD or non-CVD groupings in pairs with comparable characteristics. The matching criteria were set via a logistic regression on age and gender on CVD condition with a caliper, the maximum tolerated difference for matching, set to 0.1.

Kaplan-Meier plots were created to compare the survival status of the two groups, factoring in their treatment conditions (i.e., whether they had been treated with or without methylprednisolone). The logrank test was conducted to detect the existence of any statistical difference in survival duration. Statistical tests were performed using SPSS25 with PSM extension.

A univariate cox proportional hazards regression analysis was employed to identify the relationship between the demographic factors and vital signs and patient’s survival. In addition, a multivariate cox analysis was employed to evaluate the effects of methylprednisolone treatment when the effects of demographic factors and vital signs were controlled. The analysis was conducted using the R package of survival. All reported P values were two-sided; and all reported results bear a statistical significance with a P value less than or equal to 0.05.
